# Supplementary material for: Are carbon emissions trading and green financial instruments synergistic? -Comprehensive quantitative research based on content analysis
Source: PLoS One. 2024 Mar 7;19(3):e0298601. doi: 10.1371/journal.pone.0298601 (PMC10919601; doi:10.1371/journal.pone.0298601)
Supplement: S1 Data — (ZIP) [file pone.0298601.s001.zip › Data/地级市Z_F工作报告.zip!/╕╩╦α/╠∞╦«╩╨/└┤╫╘╣1⁄2╓┌║┼í╛┬φ┐╦╩2╛▌═°í┐_2009.docx]

各位代表:

现在,我代表市人民政府向大会报告工作,请予审议。并请政协委员和列席人员提出意见、建议。

2008年工作回顾

2008年,是我市经济社会发展进程中极不平凡的一年。我们经历了特大地震灾害和全球金融危机冲击等难以预料、历史罕见的重大挑战和考验。面对严峻形势,在市委的坚强领导下,市政府坚持以科学发展观为指导,全面落实中央和省委、省政府的各项重大决策部署,团结带领全市人民,齐心协力,顽强拼搏,一手抓抗震救灾和恢复重建,一手抓经济社会发展,较好地完成了市五届人大三次会议确定的各项目标任务,全市经济社会发展和改革开放在克服困难中取得了新的显著成绩。

抗震救灾和灾后恢复重建取得阶段性成效。去年“5·12”汶川特大地震及多次余震波及我市,给我市人民生命财产和经济社会发展造成严重损失。灾情发生后,全市上下万众一心,众志成城,开展了有力、有序、有效的抗震救灾工作,取得了重大胜利。灾后恢复重建有序推进,通过财政、金融及社会捐助等多渠道筹集抗震救灾和灾后恢复重建资金12.4亿元,当年启动实施农村灾后住房重建58908户,占农村住房重建户的77%,其中整村搬迁的114个村、7551户和分散自建的68307户住房完工率分别达到44.3%和60.4%,其他33692户农民的住房维修加固全部完成。实施校舍维修项目363项,完成维修面积25.13万平方米;实施校舍灾后恢复重建项目440项,完成面积17.2万平方米。建设过渡安置房6847套,排除安全生产隐患309处,工商企业和交通、水利、电力、通讯、供热、供水等受损基础设施的恢复重建取得较大进展。妥善安置受灾群众生活,发放“三孤”、“三无”人员生活补助金1.53亿元,救灾粮5005吨,救灾帐篷3.39万顶、被褥1.4万包、衣物2.6万包、食品14.76万件,确保了受灾群众基本生产生活和安全越冬。积极承担向陇南灾区转运救灾物资的重任,有力地保障了陇南灾区的抗震救灾工作。

经济保持平稳较快发展。全市实现生产总值226.6亿元,按可比价格计算,增长11%,其中:第一产业增加值42.3亿元,增长14.43%;第二产业增加值83.4亿元,增长10%;第三产业增加值100.9亿元,增长10.6%。非公有制经济完成增加值75.1亿元,增长11.9%。工业经济平稳增长。全市规模以上工业企业完成增加值41亿元,增长13.1%;实现利润总额2.36亿元。“10强50户”企业拉动作用明显,装备制造业主体支撑作用增强,县域工业经济发展有新起色。企业技术进步和创新能力不断提高,完成新产品研发130项,实现销售收入15亿元。“海林”牌轴承荣获“中国驰名商标”称号,28个产品获甘肃省名牌产品称号。节能减排取得成效,全年万元生产总值能耗降低4.5%,化学需氧量和二氧化硫排放量分别下降4.2%和1.07%。农业和农村经济全面发展。粮食生产获得好收成,总产103.36万吨,增长20.25%。果品、畜牧、蔬菜三大主导产业及中药材、花卉、马铃薯等特色优势产业进一步发展。水果产量62.52万吨,增长15.35%;畜禽饲养总量1468万头(只),增长14.8%;蔬菜总产130.35万吨,增长19.95%。新建及改扩建农业产业化龙头企业21个。扶贫开发扎实推进,实施整村推进项目75个,实现1万绝对贫困人口基本解决温饱、5.05万低收入人口稳定解决温饱的年度目标。劳务经济快速发展,全市劳务输转55.86万人,劳务收入27.5亿元,分别增长5.4%和31.3%。新农村建设积极推进,粮食直补、农资综合补贴、退耕还林粮食补助、良种补贴、农机具补贴等各项强农惠农政策全面落实。商贸旅游业稳步发展。全市实现社会消费品零售总额89.42亿元,增长20.7%。城乡市场建设取得新进展,甘泉、西十里物流园区建设全面启动。全市旅游业克服“5·12”地震灾害影响,接待游客389.2万人次,实现旅游综合收入13.62亿元,分别增长15.15%和20.32%。财税金融工作保持良好局面。全市完成大口径财政收入29.19亿元,增长28.25%,地方财政收入12.93亿元,同比增长18.98%;财政支出79.7亿元,增长52.85%。金融机构各项存款余额283.3亿元,较年初增长28.26%;各项贷款余额106.26亿元,较年初增长31.45%;不良贷款率较年初下降13.77个百分点。

项目工作成效显著。全市完成全社会固定资产投资143.2亿元,增长28.01%。组织实施重点建设项目107个,当年完成投资78.9亿元。工业项目建设有较大突破。实施工业项目175项,总投资69.5亿元;引进签约工业项目21个,投资总额30.23亿元。大唐甘谷电厂2×30万千瓦技改、中材天水水泥公司年产200万吨新型干法水泥生产线、甘谷祁连山水泥公司年产120万吨水泥生产线、奔马公司10万吨啤酒技改扩建、秦安长城果汁公司3万吨浓缩果汁生产、星火公司数控自动化成套设备、海林中科公司矿冶石化工程机械及军工机械轴承、天水电气传动研究所石油钻采电气传动装置产业化等一批重要工业项目顺利启动或实施。城乡基础设施项目建设取得新进展。在建交通项目748个,当年完成投资28.2亿元。天水民航恢复正常通航,宝天高速公路、天定高速公路建设积极推进,宝天高速公路市区过境段、天平铁路开工建设,国防战备公路、农村公路和运输站场等建设项目进展较快。实施市政基础设施建设项目35项,完成投资4.65亿元。藉河城区段生态环境综合治理景观工程和天河城开发、天河集中供热、青年南北路整治工程全面完工,瀛池大桥、伏羲城保护、赵家祠堂修缮保护工程等项目建设取得新进展,市商务中心、市会展中心和曹家埂经济适用房等项目前期工作进展顺利。房地产开发完成投资14.26亿元,增长10.25%。县城和小城镇建设步伐加快。创建国家园林城市、国家卫生城市、中国最佳历史文化旅游城市“三城联创”活动全面开展,市区新增公共绿地面积15.4万平方米,市容市貌和城市环境质量明显改善。实施农村重点基础设施项目50项,完成各类造林19.66万亩,新修梯田13.38万亩,治理水土流失面积200平方公里,修建各类小型水利工程200处,新增有效灌溉面积1.6万亩、节水灌溉面积1.88万亩。关中—天水经济区规划编制和扩内需、促增长项目申报争取工作进展较好。积极配合国家发改委编制了《关中—天水经济区发展规划》,完成了《关中一天水经济区天水市发展规划》的编制。认真贯彻党中央、国务院关于进一步扩大内需促进经济平稳较快增长的政策措施,围绕国家投资重点,抓紧项目的筛选、论证、上报工作,争取扩大内需项目126个,落实国家投资3.81亿元。

改革开放深入推进。国企改革取得新进展,长材厂等5户企业基本完成破产重组任务,岐黄药业有限公司完成股权多元化改制,轴仪厂和焦化厂破产重组启动实施,岷山厂等7户企业的改制工作取得实质性进展。农村综合改革稳步推进,在稳定和完善农村基本经营制度的基础上,探索了土地承包经营权流转的多种形式和有效途径;积极推进集体林权制度改革,清水县被列为全省集体林权制度改革和支农资金整合试点县,秦安县“一册明、一折统”的支农资金管理经验在全省推广。财政改革逐步深化,市级国库集中支付制度改革全面完成,部门预算管理扩大到县区,财政监管和审计监督不断加强。金融改革积极推进,“招行引资”成效明显,成立了兰州银行天水分行、中国邮政储蓄银行二级分行、秦州农村合作银行和秦安众信村镇银行,金融服务体系逐步健全。机构编制工作进一步加强,事业单位岗位设置、人员公开招聘、编制实名制管理工作全面启动。对外开放深入推进,主动加强与国内外知名企业,特别是关中地区的经济技术交流与合作,天水星火机床有限责任公司成功收购法国索玛(SOMAB)公司、参股德国亨利安(WMH)公司,武汉凯迪生物质能源等重大项目落户我市。招商引资成效显著,全年签约各类招商引资和战略合作项目166项,总投资64.65亿元,实到引资32.42亿元,增长37%。进一步调整外贸出口产品结构,努力扩大对外贸易,全市进出口总额达到1.18亿美元,增长30%。

社会事业全面发展。农村义务教育经费保障机制改革政策得到较好落实,“两基”成果进一步巩固提高,教育教学质量明显提升,全市高考文理科二本以上上线率达到18.18%,提高2.34个百分点。职业教育加快发展,中职招生2.32万名,毕业生就业率稳中有升。高等教育、成人教育、民办教育、学前教育和特殊教育稳步发展。科技创新能力不断增强,围绕十大科技专项,组织实施市以上科技项目345项,取得科技创新成果206项,申请专利210件。天水农业高新技术示范园区晋级省级农业科技示范园区。科技推广应用步伐加快,引进示范推广新品种、新技术98项。院地院企科技合作不断加强。特色文化大市建设积极推进,规划建设了一批城乡文化基础设施,开展了丰富多彩的文艺演出活动,举办了多种形式的书画、摄影、民间工艺制品、非物质文化遗产保护等主题展览,城乡群众文化生活更加活跃。广播电视“村村通”、无线覆盖、数字电视、农村电影放映等工程建设进展顺利。以迎奥运为主题的全民健身活动广泛开展,竞技体育水平不断提高。医疗卫生事业加快发展,传染病防控、计划免疫等公共卫生工作进一步加强,城乡公共卫生服务体系不断完善,新型农村合作医疗制度全面落实。人口和计划生育年度目标任务完成,全市人口出生率、自然增长率分别为12.78‰和6.93‰,农村计划生育率达到87.18%。国土资源管理、统计、人防、消防、气象、档案(地方志)、外事侨务、老龄、妇女儿童、残疾人和慈善等工作都有新进步。

人民生活持续改善。城镇居民人均可支配收入和农民人均纯收入分别达到9050元和2148元,增长8.8%和19.1%。全面落实扶持就业与再就业的各项优惠政策,深入开展全民创业活动,努力拓宽就业渠道。全市新增城镇就业5.8万人,下岗失业人员再就业2.57万人,困难人员再就业1.1万人,期末城镇登记失业率为2.85%,低于控制目标1.15个百分点。社会保障体系不断完善,城镇职工养老、失业、基本医疗、工伤和生育保险覆盖范围进一步扩大,各项社会保险金
